# Predicted Brain Age After Stroke

**DOI:** 10.3389/fnagi.2019.00348

**Published:** 2019-12-10

**Authors:** Natalia Egorova, Franziskus Liem, Vladimir Hachinski, Amy Brodtmann

**Affiliations:** ^1^Division of Behavioural Neuroscience, The Florey Institute of Neuroscience and Mental Health, Melbourne, VIC, Australia; ^2^Melbourne School of Psychological Sciences, University of Melbourne, Melbourne, VIC, Australia; ^3^University Research Priority Program Dynamics of Healthy Aging, University of Zurich, Zurich, Switzerland; ^4^Department of Clinical Neurological Sciences, Western University, London, ON, Canada; ^5^Department of Epidemiology and Biostatistics, Schulich School of Medicine and Dentistry, Western University, London, ON, Canada

**Keywords:** age prediction, structural magnetic resonance imaging, stroke, chronological age, brain age

## Abstract

Aging is a known non-modifiable risk factor for stroke. Usually, this refers to chronological rather than biological age. Biological brain age can be estimated based on cortical and subcortical brain measures. For stroke patients, it could serve as a more sensitive marker of brain health than chronological age. In this study, we investigated whether there is a difference in brain age between stroke survivors and control participants matched on chronological age. We estimated brain age at 3 months after stroke, and then followed the longitudinal trajectory over three time-points: within 6 weeks (baseline), at 3 and at 12 months following their clinical event. We found that brain age in stroke participants was higher compared to controls, with the mean difference between the groups varying between 3.9 and 8.7 years depending on the brain measure used for prediction. This difference in brain age was observed at 6 weeks after stroke and maintained at 3 and 12 months after stroke. The presence of group differences already at baseline suggests that stroke might be an ultimate manifestation of gradual cerebrovascular burden accumulation and brain degeneration. Brain age prediction, therefore, has the potential to be a useful biomarker for quantifying stroke risk.

## Introduction

The chronological age of an individual is not necessarily the same as their physiological—or “biological”—age. The trajectory of physiological aging is affected by individual differences in genetics and life style. For example, different people will have different amounts of brain volume loss in late life, depending on their education level, lifestyle, and level of physical activity (Steffener et al., 2015). Brain health can be measured, meaning that brain age could be quantified.

The difference between chronological and brain age can be a sensitive predictor and a clinically relevant biomarker of different disorders, especially ones associated with aging. A number of authors have shown the utility of using the difference between chronological and predicted brain age to identify cognitive impairment (Liem et al., 2017), Alzheimer’s disease (AD; Franke et al., 2010), predict mortality (Cole et al., 2018), and the conversion from mild cognitive impairment to AD (Gaser et al., 2013).

Stroke incidence disproportionately increases with age (Go et al., 2013). Older age is a risk factor for stroke; it also worsens outcomes after stroke (Sohrabji et al., 2013). The proposal that stroke is specifically associated with accelerated brain aging prior and after stroke has been made by several authors. Neuroimaging markers of brain aging, such as smaller hippocampal and total brain volumes, and increased white matter hyperintensity load, have been linked to both, vascular brain injury prior to the stroke event (Seshadri et al., 2004; Knopman and Hooshmand, 2017; Werden et al., 2017) and continued brain atrophy and neurodegeneration (Knopman et al., 2009; Kooi Ong et al., 2017). For acute stroke, it has been estimated that during stroke or transient ischemeic attack (TIA), compared with the normal rate of neuronal loss in brain aging, the ischemic brain ages 3.6 years each hour without treatment (Saver, 2006). Furthermore, the effect of stroke on cognitive function has been shown to be equivalent to aging 7.9 years (Levine et al., 2015).

Previous studies have not directly compared brain age to chronological age in stroke. In this study, we applied age-predicting models trained on a large cohort of healthy participants and validated in several independent datasets (Liem et al., 2017), to investigate differences between brain and chronological age in stroke and healthy participants at 3 months post-stroke, as well as the longitudinal trajectory of brain age (at 6 weeks, 3 months and 12 months), with the intention of assessing feasibility of age prediction in stroke. We hypothesized that although the groups of stroke and control subjects were not significantly different in their chronological age, stroke participants’ brains would have an estimated brain age greater than controls.

## Materials and Methods

### Participants

This work was based on data from the Cognition and Neocortical Volume after Stroke (CANVAS) study (Brodtmann et al., 2014). Ischemeic stroke patients were recruited within 6 weeks of their event from the Stroke Units at three Melbourne hospitals: Austin Hospital, Box Hill Hospital, and the Royal Melbourne Hospital. Each hospital’s ethics committee approved the study in line with the Declaration of Helsinki. Patients had clinically and radiologically confirmed first-ever or recurrent ischemic stroke in any vascular territory. The severity of participants’ stroke was assessed with the National Institutes of Health Stroke Scale (NIHSS) examination performed at hospital admission. Healthy control participants were recruited from the database of volunteers who had previously undertaken MRI scanning or volunteered for studies at the Florey Institute of Neuroscience and Mental Health. Stroke and control participants had no history of dementia or neurodegenerative disorders, major psychiatric illnesses or substance abuse problems. Participants’ demographic and clinical data are described in Table 1.

**Table 1:** Demographic and clinical variables by group.

Variable	Stroke	Control	Stroke vs. Control (*p*-values)	Test (2-tailed)
*N*	135	40	n/a	n/a
Sex (*N* female)	41	15	0.39	Chi-Square
N right-handed	10	4	0.52	Fisher exact test
NIHSS baseline (Median, range)	2 (0–15)	n/a	n/a	n/a
Years of education (Mean, SD)	12.66 (3.66)	15.48 (4.53)	<0.001	*t*-test
Age (years, Mean, SD)	67.41 (13.01)	68.65 (6.64)	0.49	*t*-test
Total intracranial volume, ml (Mean, SD)	1,518 (125)	1,502 (165)	0.47	*t*-test
Lesion volume, ml (Mean, SD)	10 (30)	n/a	n/a	n/a
BMI baseline	27.74 (4.72)	26.55 (3.81)	0.11	*t*-test
Smoking, pack-years (Median, range)	1 (0–120)	0 (0–50)	0.051	Mann-Whitney test
Family history of stroke (*N*)	42	15	0.44	Chi-Square
High cholesterol (*N*)	62	14	0.22	Chi-Square
Hypertension (*N*)	85	17	0.021	Chi-Square
Atrial fibrillation (*N*)	33	1	0.001	Fisher exact test
T2DM (*N*)	34	4	0.049	Fisher exact test
Stroke laterality (*N*)		n/a	n/a	n/a
Left	50			
Right	82			
Bilateral	3			
Stroke type, Oxfordshire classification (*N*)		n/a	n/a	n/a
LACI	19			
PACI	70			
POCI	44			
TACI	2			

*N, number; SD, standard deviation; NIHSS, National Institutes of Health Stroke Scale; BMI, Body Mass Index; T2DM, Type 2 Diabetes Mellitus; LACI, lacunar circulation infarcts; PACI, partial anterior circulation infarcts; POCI, posterior circulation infarcts; TACI, total anterior circulation infarcts*.

### Imaging Data Acquisition and Analysis

All images were acquired on a Siemens 3T Tim Trio scanner (Erlangen, Germany) with a 12-channel head coil. The same scanner was used for participants recruited from different hospitals. As part of an ongoing longitudinal study (Brodtmann et al., 2014), participants were assessed within 6 weeks, then again at 3 and 12 months after their stroke. A high-resolution anatomical MPRAGE was collected (volume of 160 sagittal slices with 1 mm isotropic voxels, TR = 1,900 ms, TE = 2.55 ms, 9° flip angle, 100% field of view in the phase direction and 256 × 256 acquisition matrix). A high-resolution 3D SPACE-FLAIR image was acquired (with 160 1 × 0.5 × 0.5 mm sagittal slices, TR = 6,000 ms, TE = 380 ms, 120° flip angle, 100% field of view in the phase direction and 256 × 254 acquisition matrix).

We automatically estimated structural volumes using FreeSurfer 5.3^1^ at each time-point separately. Tissue segmentations for individual subjects were visually inspected and corrected. Total intracranial brain volumes for each participant were estimated.

Lesions were manually traced on the high-resolution FLAIR image. A stroke neurologist (AB) visually inspected and verified the manually traced images. A binary lesion mask was created and normalized to the MNI152 template using the Clinical Toolbox SPM extension (Rorden et al., 2012). Lesion volumes were computed from the masks using FreeSurfer. Lesion overlap images were prepared using MRIcron software (Rorden et al., 2007; Supplementary Figure S1).

### Age Prediction

Models were trained to predict age based on measures of cortical anatomy (cortical thickness, cortical surface area, subcortical volumes) as described in Liem et al. (2017). Native surface models for cortical thickness and surface area computed in FreeSurfer were transformed into the fsaverage4 standard space and the two hemispheres data were concatenated. Volumes of subcortical regions and measures of global volume were extracted from the “FreeSurfer aseg.stats” files. After extracting the feature vectors for each subject and for each of the different anatomical measures, predictions were stacked *via* a Random Forest model, producing the stacked anatomy measure including cortical thickness, cortical surface area, and subcortical volumes.

Linear support vector regression model (SVR) was used to predict age from structural neuroimaging data. The training sample was a subsample of the LIFE-Adult-Study [Leipzig Research Centre for Civilization Diseases (LIFE), life.uni-leipzig.de (Loeffler et al., 2015)]. Specifically, it included randomly selected community-dwelling volunteers between 20 and 80 years who had MRI assessment and neuropsychological testing performed and who were found to have no objective cognitive impairment based on standardized scores of cognitive performance (OCI norm in Liem et al., 2017), *N* = 1,166, sex: 566 female, age: *M* = 59.1, standard deviation (SD) = 15.2. The MRI data for the sample were acquired on a 3T Siemens Trio scanner with a 32 channel head coil. High-resolution T1 images were acquired with an MPRAGE sequence with 1 mm isotropic voxels, 176 slices, TR = 2,300 ms, TE = 2.98 ms, TI = 900 ms. Generalizability of the model has been previously tested and validated in another sample [the Enhanced Nathan Kline Institute Rockland sample (Nooner et al., 2012)] from a different country, with data from a different scanner, with a different acquisition protocol and subjects.

Predictive analyses were performed using the python- and scikit-learn based methods implemented in BARACUS 0.9.4 (Brain-Age Regression Analysis and Computation Utility Software^2^), as described in detail in Liem et al. (2017).

### Statistical Analyses

To evaluate age prediction quality with different measures of anatomy, we performed Pearson correlations between chronological and predicted age estimated for each brain measure for the whole cohort (stroke and control participants combined) and for each group. In addition, we report a mean absolute error (MAE) and coefficients of determination, *R*^2^ calculated using the *R*^2^_score function implemented in Scikit-learn. Note that the *R*^2^ here represents a comparison of the values predicted by the model against the values predicted by a dummy model. The value of *R*^2^ can be zero if the model predicts the expected value disregarding the input features, or negative if the model performs arbitrarily worse. To evaluate the relationship between stroke lesions and predicted age, we computed a correlation between lesion volume and predicted age, accounting for the NIHSS at baseline.

As typically done in studies of predicted age, to compare stroke and control groups, we calculated the so-called brain aging score by subtracting chronological age from predicted age for all participants. We then performed between-group ANCOVAs for each brain measure based on the data from 3 months (controls: *N* = 40, stroke: *N* = 124), accounting for years of education, as it has been previously shown to be associated with brain age (Steffener et al., 2015). To evaluate the relationship between stroke lesions and brain scores, we performed a correlation between lesion volume and predicted age, accounting for the NIHSS at baseline. For all analyses, we performed 2-tailed tests and the results were considered significant at *p* < 0.05.

Finally, to investigate whether there were any differences in brain aging over time, we performed a longitudinal analysis on all 175 subjects, even if some participants did not have data for all time points, using a mixed linear regression, as implemented in matlab *fitlme* function. We designed the mixed-effect model based on a maximal random effects structure, with factors Time (baseline, 3 and 12 months) and Group (control vs. stroke) and years of education as a covariate. A random intercept and a random (Time) slope varied by subject were also included in the *lme* model. The random intercept and slope were modeled with a possible correlation between them.

## Results

### Behavioral Results

The total of 175 participants took part in the CANVAS study at one or more time points (6 weeks, 3 months, 12 months): see Table 1 for the description of the whole cohort and Supplementary Figure S1 for the lesion overlap map in stroke participants. Predicted age data were obtained from participants who completed a structural MRI scan at least for one time point. The sample size varied for each time point: *N* = 125 at 6 weeks, *N* = 164 at 3 months, and *N* = 151 at 12 months. For more in-depth cross-sectional analysis we used data at 3 months, given the highest number of available participants and the fact that 3 months after stroke is considered an early but relatively stable stage following stroke. For longitudinal analyses, we used data from all 175 participants, even though some of them had missing data (all three time points were available for 39 control and 65 stroke participants).

### Age Prediction Performance With Different Brain Measures

**Figure 1:**
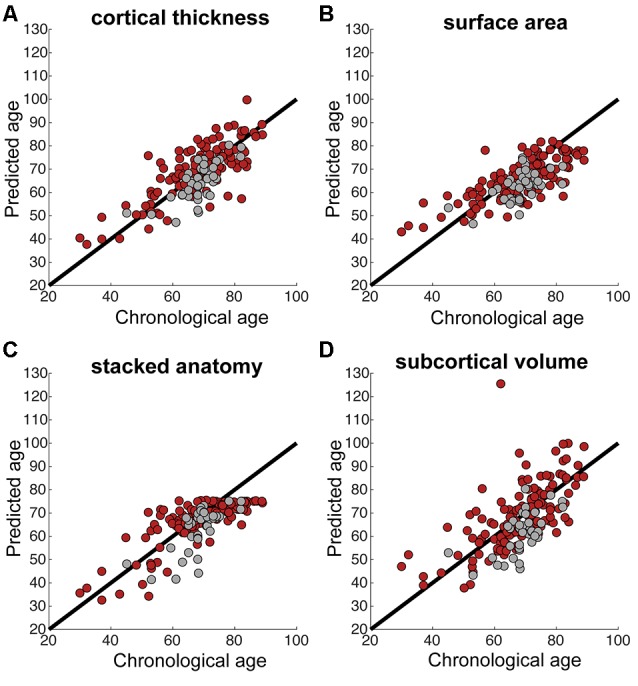
**(A–D)** Correlations between chronological and predicted age for each of the brain measures. Black line shows the perfect correlation, red circles represent stroke participants, gray circles represent control participants. Note the tendency for underestimated predicted age in control participants in most measures (gray circles below the black line).

**Figure 2:**
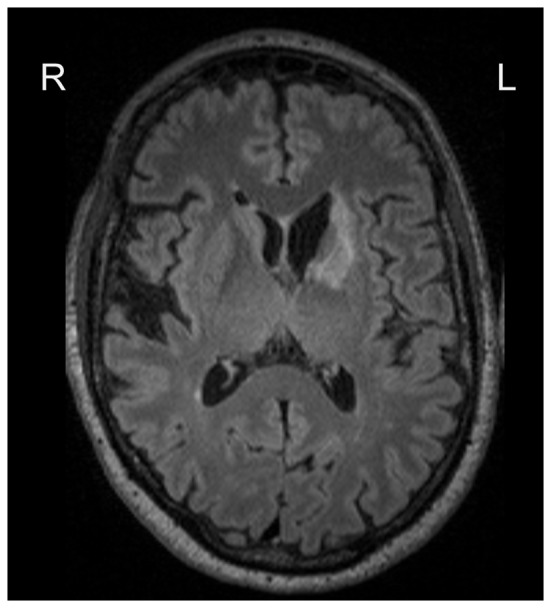
Lesion location for the participant with an outlier age prediction based on the subcortical volume.

We calculated correlations between predicted and chronological age, using data at 3 months, see Figure 1 for the correlation plots, showing controls and stroke participants separately (correlations by group are shown in Table 2). There were significant correlations for all measures: stacked anatomy (*r* = 0.75, *p* < 0.001; coefficient of determination *R*^2^ = 0.48, MAE = 6.1), cortical thickness (*r* = 0.75, *p* < 0.001; *R*^2^ = 0.5, MAE = 5.9), surface area (*r* = 0.71, *p* < 0.001; *R*^2^ = 0.38, MAE = 6.8), and subcortical volume (*r* = 0.63, *p* < 0.001, *R*^2^ = 0.03, MAE = 8.3). Note that for the subcortical volume measure, when 1 outlier (predicted age of 125) was removed the correlation was *r* = 0.69, *p* < 0.001, *R*^2^ = 0.18). The location of the striatocapsular infarction for this participant is shown in Figure 2. Note also that in the group results shown in Table 2 some *R*^2^ values are negative, especially for the control group. The chronological age in our sample is narrowly centered around 70 years; there is a known negative bias in predicting older age by the model (Le et al., 2018; Liang et al., 2019) where predicted age is underestimated. In the stroke group, where the predicted brain age appears older, it is less of a problem, but in the controls this systematic underestimation results in “poor” performance of the model due to the shift in absolute predicted values that are reflected in the negative *R*^2^ scores, despite a reasonable correlation *r* and MAE.

**Table 2:** Correlations between biological and predicted age for each measure and group.

Measure	Stroke				Control			
	*r*	*p*-value	*R*^2^	MAE	*r*	*p*-value	*R*^2^	MAE
Stacked anatomy	0.79	<0.001	0.60	6.02	0.71	<0.001	−0.68	6.47
Cortical thickness	0.78	<0.001	0.56	6.01	0.71	<0.001	−0.17	5.56
Cortical surface area	0.75	<0.001	0.50	6.66	0.56	<0.001	−0.77	7.34
Subcortical volume	0.66	<0.001	0.12	8.13	0.61	<0.001	−1.57	8.87

### Comparison Between Stroke and Control Participants in Predicted Age

**Figure 3:**
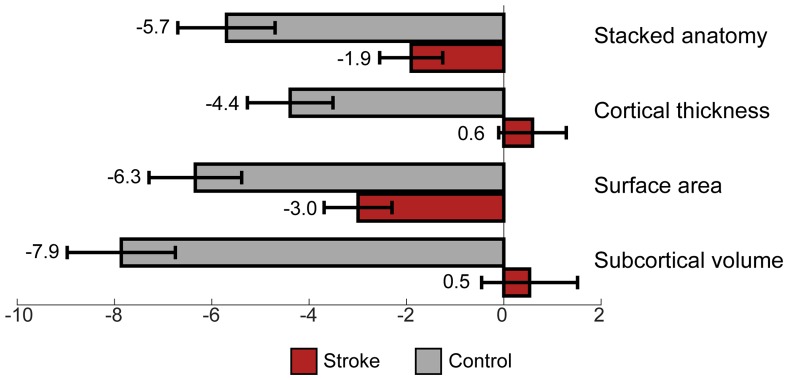
Brain aging score (chronological minus predicted age) for control vs. stroke participants, at 3 months after stroke.

A comparison of brain aging scores between control and stroke participants using 3 months data showed a significant difference, accounting for years of education for all measures, see Table 3. The comparison of brain aging scores between the groups computed from stacked anatomy revealed the mean brain aging score in the control group was Mean (SD) = −5.7 (6.4) and for stroke group −1.9 (7.2); for cortical thickness the values were −4.4 (5.6) and 0.6 (7.7); for surface area −6.3 (6.0) and −3.0 (7.8), for subcortical volume −7.9 (7.0) and 0.5 (11.0), respectively (see Figure 3).

**Table 3:** Differences in brain aging scores between control and stroke participants, controlling for years of education.

Measure	Average difference in predicted age (Stroke > Control), years (SD)	Statistical results
Stacked anatomy	3.87 (1.34)	*F*_(1,161)_ = 8.385, *p* = 0.004
Cortical thickness	5.26 (1.38)	*F*_(1,161)_ = 14.498, *p* < 0.001
Cortical surface area	4.17 (1.39)	*F*_(1,161)_ = 9.079, *p* = 0.003
Subcortical volume	8.73 (1.93)	*F*_(1,161)_ = 20.424, *p* < 0.001

We also specifically investigated the relationship between lesion volume and stroke severity on the age prediction (predicted age and brain scores) at 3 months. No measures (stacked anatomy, cortical thickness, cortical surface area, subcortical volume) were significantly associated with lesion volume, accounting for NIHSS at baseline in either predicted age or brain scores.

### Longitudinal Analysis of Predicted Age Between Stroke and Control Participants

Longitudinal analysis performed on all 175 available subjects with missing data in some of the time points, using mixed linear regression, revealed no significant main effect of time or time by group interaction, suggesting no change in predicted age over 1 year in either of the groups. However, this analysis replicated the main effect of group in each of the measures [stacked anatomy (Estimate = 3.54, SE = 1.34, *t* = 2.63, *df* = 433, *p* = 0.008, 0.9–6.2); cortical thickness (Estimate = 5.1806, SE = 1.35, *t* = 3.8376, *df* = 433, *p* < 0.001, 2.5–7.8); surface area (Estimate = 4.5844, SE = 1.3985, *t* = 3.278, *df* = 433, *p* = 0.001, 1.8–7.3); subcortical volume (Estimate = 9.0133, SE = 1.886, *t* = 4.779, *df* = 433, *p* < 0.001, 5.3–12.7)].

## Discussion

In this study, we predicted participants’ brain age based on various brain measures, using an existing brain prediction model implemented in BARACUS (Liem et al., 2017). We compared brain aging scores in stroke and control populations at 3 months, then longitudinally over a period of a year. Although stroke and control participants were matched on their chronological age, their brain age was significantly different at all time points. In the absence of chronological age differences, brain aging score could be a more sensitive measure of aging and should be investigated as a biomarker of stroke risk and predictor of stroke recovery in future studies.

### Prediction of Age in a Stroke Cohort

Previous studies using the prediction model applied here (Liem et al., 2017) showed that using different brain measures for age prediction yield similar results, with the stacked anatomy measures producing the smallest prediction error. In this study, the most accurate age prediction (based on the whole cohort correlation between chronological and predicted age) was using stacked anatomy and cortical thickness measures. Age prediction based on cortical thickness was also found to be more accurate than surface area prediction in a previous study (Liem et al., 2017). Note that stacked-anatomy predictions did not exceed a value of around 75 years in this study (see Figure 1). This plateau effect, which can also be seen in Figure 2 of Liem et al. (2017) is due to the nature of the stacking approach. It uses Random Forest models which cannot extrapolate beyond the age range of the training set.

Overall, age prediction in stroke participants was feasible and produced fairly accurate results for all measures (a correlation of 0.7 between chronological and predicted age). On average, the predicted age results were not significantly associated with lesion extent. However, one outlier showing predicted age of 125 based on the subcortical volume measure was observed in a stroke participant with a rather extensive subcortical lesion. At the same time, the possibility to predict brain age based on cortical, subcortical measures or their combination, overcomes the potential problem arising from specific lesion distribution. For example, for this participant with a chronological age of 62, the other derived measures showed predicted age of 75 (stacked anatomy), 77 (cortical thickness) and 68 (surface area), which are all within the normal range of predicted values and can be used as biomarkers of age.

Note that we applied this prediction model in a group of older ischemeic stroke patients, however, the same method was used for stroke and healthy participants alike, and no specific limitation on the age of the group was set, making the model versatile and potentially useful for different stroke types and age cohorts.

### Differences Between Stroke and Control Participants in Brain Age

We observed consistently lower predicted age in control participants, compared to stroke subjects. Typically, the age prediction model that we used (Liem et al., 2017) tends to overestimate age in younger adults and underestimate age in older adults. A previously reported prediction error (underestimation for older adults) for this model in normal population is 4.83–7.29 years, depending on the measure used. With controls in our study we observed brain aging scores between −4.39 and −7.86 years. Although in our study we cannot calculate prediction error and disentangle it from the brain age estimates, the predicted age values are within the same range (i.e., underestimated) for our control population.

The brain aging scores for stroke participants were between −3 years (underestimation) and 0.6 years (overestimation). This means that stroke participants’ brain age was estimated as higher in both relative and absolute terms; i.e., compared to our control subjects and compared to the previous normal population showing underestimation. Figure 1 demonstrates that control participants’ age in our study is underestimated (gray circles below the black line), whereas stroke participants’ age is not.

Although there were no differences between the groups in chronological age, there were more participants under 45 in the stroke sample. To rule out the possibility that systematic over-estimation in younger stroke participants would explain higher brain scores in the stroke group, we repeated both cross-sectional and longitudinal analyses excluding participants under 45 (*N* = 8). All the reported results remained significant.

Previously, Liem et al. (2017) found that the discrepancy between predicted age and chronological age also captured the degree of cognitive impairment, with the strongest differences in brain aging between the participant groups observed in the model using subcortical data. In our study, subcortical volume data also produced the biggest numerical between-group difference in brain aging scores. The measure of cortical thickness also showed a strong group effect in both cross-sectional and longitudinal analyses. Note that both subcortical volume and cortical thickness measures showed a positive mean brain aging score (higher predicted than chronological age), whereas surface area measure showed a negative score. Our results are consistent with the finding that cortical thickness-based prediction is better than surface area results (Hogstrom et al., 2013).

### Rate of Brain Aging

Previous studies have shown that the brain continues to show signs of neurodegeneration after stroke, such as thalamic volume decline (Tamura et al., 1991; Ogawa et al., 1997; Yassi et al., 2015). Contrary to our expectation, there were no differences between stroke and healthy control participants in the *rate* of brain aging. This could be due to a number of reasons.

It is possible that we lacked sensitivity to observe an effect over just 1 year. No main effect of time was observed, even in control participants, although raw predicted age on average increased in all participants over time, suggesting some longitudinal sensitivity. The brain segmentations that were used here were based on a cross-sectional FreeSurfer approach, which is less sensitive to longitudinal changes. Our analysis focused on the brain aging score, which includes not only information about the age (and its progression over time) but also any changes in brain state. During the first year after stroke there is an ongoing, large-scale brain reorganization associated with recovery and decline. It is, therefore, possible that any changes over time were obscured by these complex, non-linear, and within-group individual differences in recovery. The measures we used were whole-brain or whole subcortical measures. Brain decline after stroke is often localized or network-specific (Wu et al., 2008; Schaapsmeerders et al., 2015; Egorova et al., 2018; Veldsman et al., 2018), so that at a global whole-brain level, minor longitudinal changes were not observable using the current model of age prediction. Finally, it is possible that even with 175 subjects, we lacked the power to observe the interaction effect.

### Implications and Future Directions

The lack of longitudinal changes poses an interesting question on the nature of the group difference in brain age at baseline. On the one hand, the presence of an acute lesion would affect the brain age negatively, as suggested by Saver (2006) postulating the brain decline rate of about 3.6 years per hour in untreated stroke. However, we did not observe any significant relationship between biological age and stroke lesions or stroke severity. Furthermore, despite some expected post-stroke neurodegeneration in the first year after stroke, such as in the hippocampi and thalami, no significant decline and sustained difference between stroke and control participants were observed over the course of 1 year. This suggests that in principle, the measured brain age at baseline, which was obtained as early as 6 weeks after stroke, was the reflection of longer-term brain damage accumulated possibly even before the stroke event itself. Note also that although our control and stroke groups were matched on chronological age, they differed significantly in their level of education, as well as a number of vascular risk factors. While we attempted to account for the education differences, which have been previously shown to be associated with brain age (Steffener et al., 2015), vascular risk factors, such as hypertension, type 2 diabetes, atrial fibrillation often accompany stroke and difficult to dissociate from the stroke diagnosis. In other words, it is possible that vascular risk factors, gradual cerebrovascular burden accumulation and brain degeneration prior to stroke influenced the estimates of brain age analyzed here. If so, future studies could investigate brain age as a sensitive predictor of stroke, compared to chronological age.

In addition to assessing stroke risk, brain age could be a sensitive biomarker of recovery. It has been shown that biological age estimated based on DNA methylation is a better predictor of outcome after acute stroke than chronological age (Soriano-Tárraga et al., 2017). Future studies could focus on identifying the utility of brain age estimates on stroke outcome prediction and compare the biological age derived by imaging to the biological age obtained with the DNA methylation technique.

Finally, the finding that there is a significant difference between chronological and biological age between stroke and control participants has important implications for clinical trials. Future clinical studies could include matching controls and stroke patients on *biological age*. This may allow the detection of treatment effects that might be otherwise masked by accelerated structural brain aging associated with stroke and cerebrovascular risk factors.

The data that support the findings of this study are available on reasonable request from the corresponding author. The data are not publicly available as CANVAS is a prospective, “live” study, with an expected completion of data acquisition in mid-2020 for the 5-year scanning timepoint. All requests for raw and analyzed data will be reviewed by the CANVAS investigators to determine whether the request is subject to any intellectual property or confidentiality obligations.

## Data Availability Statement

The datasets generated for this study are available on request to the corresponding author.

## Ethics Statement

The studies involving human participants were reviewed and approved by Austin Hospital, Box Hill Hospital, and the Royal Melbourne Hospital. The patients/participants provided their written informed consent to participate in this study.

## Author Contributions

NE, FL, VH and AB: conception, design of the study and manuscript preparation. NE and FL: acquisition and analysis of data. NE: figure preparation.

## Conflict of Interest

The authors declare that the research was conducted in the absence of any commercial or financial relationships that could be construed as a potential conflict of interest.

## Footnotes

^1^http://surfer.nmr.mgh.harvard.edu/

^2^https://doi.org/10.5281/zenodo.843491

## Supplementary Material

FIGURE S1 Stroke lesion overlap at 3 months.
