# Supplementary figures and images for: Predicted Brain Age After Stroke
Source: Front Aging Neurosci. 2019 Dec 10;11:348. doi: 10.3389/fnagi.2019.00348 (PMC6914736; doi:10.3389/fnagi.2019.00348)

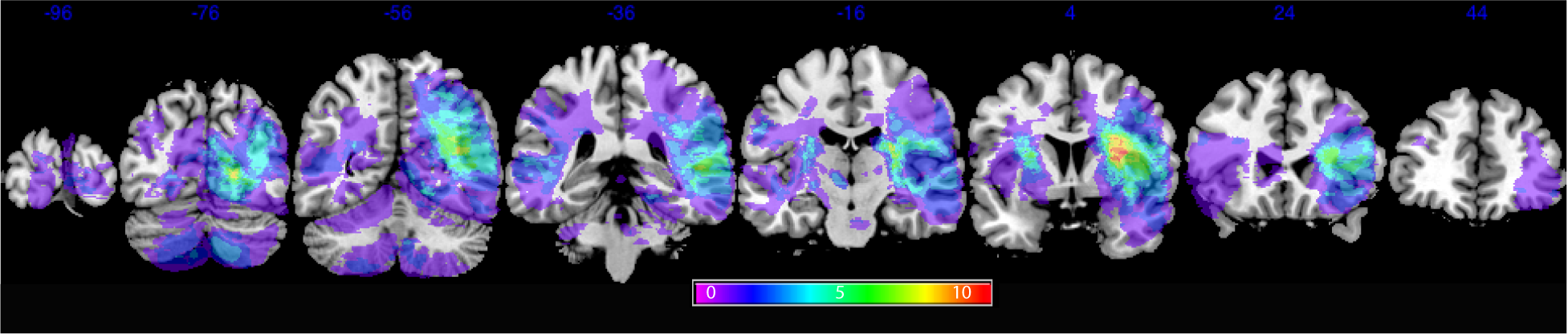

Supplement: FIGURE S1 — Stroke lesion overlap at 3 months. [file Image_1.tif]
